# Generation of Randomly Inclined Fibers in the Representative Volume Element for Predicting the Elastic Modulus of Fiber-Reinforced Polymer Composites

**DOI:** 10.3390/polym17172300

**Published:** 2025-08-25

**Authors:** Menglong Shao, Songling Xue

**Affiliations:** 1 School of Transportation Science and Engineering, Beihang University, 37 Xueyuan Road, Beijing 100191, China; mlshao@buaa.edu.cn; 2 School of Civil and Ocean Engineering, Jiangsu Ocean University, 59 Cangwu Road, Lianyungang 222005, China

**Keywords:** unidirectional fiber reinforced polymer composites, representative volume element, periodic boundary conditions, randomized algorithm, fiber inclination, fiber volume fraction

## Abstract

The representative volume element (RVE) is frequently used to forecast the mechanical properties of composites, where the distribution of fibers plays a significant role. This paper proposes a new RVE modeling method for unidirectional fiber-reinforced polymer (UD-FRP) composites, which takes into account the random distribution of fiber positions and inclinations. The fiber inclination in the RVE is normally or uniformly distributed. The suggested RVE model was validated using static tests and the fiber structure observed by micro-computed tomography (CT). The effects of fiber volume fraction and maximum fiber inclination on the elastic properties were investigated based on the proposed RVE model. The results indicate that the prediction of transverse properties is considerably impacted by fiber inclination in RVE, with uniformly distributed inclination having a more significant influence than normally distributed inclination. For the transverse Young’s modulus of UD-FRP, the predicted results of the proposed model and the models in the literature differed from the experimental results by 0.30% and 11.45%, respectively. For the in-plane shear modulus of UD-FRP, the predicted results of the proposed model and the models in the literature differed from the experimental results by 1.65% and 8.44%, respectively. Moreover, the fiber volume fraction has a significant effect on the elastic properties, and the maximum inclination of the fibers has a significant effect on the elastic properties except for the longitudinal Poisson’s ratio. The proposed RVE model in this paper can predict the elastic properties of composites more accurately.

## 1. Introduction

Continuous fiber-reinforced polymer (FRP) composites have been widely used in construction, aerospace, transportation, and medical fields in recent years due to their high strength-to-density ratio, corrosion resistance, and fatigue resistance [1,2,3,4]. Continuous fiber composites are fabricated from unidirectional (UD) FRP through lamination or winding processes. The properties of UD-FRP significantly influence the mechanical behavior of composite structures. Therefore, understanding the properties of UD-FRP is of significant engineering importance [5,6,7]. Fibers cause FRP to exhibit heterogeneity and anisotropy, and the manufacturing process, component properties, and geometric configuration can all lead to uncertainty in FRP properties at different scales [8,9,10]. To address this issue, the homogenization theory was proposed to predict the equivalent properties of FRP [11,12,13]. The homogenization method based on the representative volume element (RVE) can consider changes in the microscopic geometry of polymers and fibers, and is therefore widely used [14,15]. The selection of RVE, i.e., the layout of fibers and matrix, affects the prediction of FRP properties, and therefore, the selection of an appropriate RVE is a critical issue for FRP design.

In recent years, there has been a focus on the RVE selection for UD-FRP. The effects of different RVE fiber layouts, including square, diamond, hexagonal, and random distributions, on the property prediction were investigated [16], where all the fibers with equal diameters are oriented in the same direction. It is found that the effect of the layout of these fibers on the prediction of elastic properties was not significant. Recent studies have also focused on methods for generating randomly distributed fibers in RVE. A new algorithm for generating randomly distributed fibers was developed [17], which takes into account differences in fiber diameter and fibers crossing RVE boundaries. It is found that the generated RVEs are in good agreement with the actual fiber structure, and the random distribution of fiber diameters has little effect on the property prediction. Similarly, an RVE generation method for high fiber volume fraction was developed based on the stochastic event-driven molecular dynamics simulation [18]. Subsequently, the elastic properties of UD-FRP with fiber volume fractions between 10 and 80% were calculated using RVE, and the predicted results were found to be consistent with the experimental results. The differences in predicting elastic properties between ideal and random distributions of fibers were compared, where randomly distributed fibers were obtained based on a random perturbation algorithm and a perfect elastic collision algorithm [19]. It is found that the transverse tensile modulus predicted by RVE with rectangular and ortho-hexagonal fiber layouts is higher and lower than that predicted by RVE with randomly arranged fibers, respectively. From these studies, it is evident that the layout of fibers in the cross-section has little effect on the property prediction. However, in the existing RVE of UD-FRP, all the fibers are aligned along the same direction, which is not consistent with the actual UD-FRP. The initial inclination defects in the longitudinal direction of the fibers are not considered in the scale of the RVE. The prediction of equivalent elastic, viscoelastic, and plastic properties of UD-FRP using RVEs that do not take into account the fiber inclination may cause large deviations.

In order to accurately predict the properties of UD-FRP using RVE, fiber inclination has to be taken into account. This fiber inclination has been observed by the author in this paper and also in the experiments in the literature, which provides an unwavering opportunity for the development of a new RVE model for UD-FRP. A cubic RVE model of carbon FRP with a side length of 50 μm was developed based on micro-CT [20], where the fiber diameter was 5.2 μm. It is found that the unidirectional fibers have significant random inclination but no waviness defects. A method for splitting and extracting single fibers was proposed for a hybrid FRP with a high fiber volume fraction [21]. It is found that the extracted fibers are significantly inclined, and the compressive strength estimated from the average inclination of the fibers is in agreement with the experimental results. There were many similar studies [22] on observing the fiber structure of FRP with micro-CT, where initial inclination defects of the fibers were observed. However, the experimentally observed fiber inclination was not considered in the existing RVE model of UD-FRP. These experimental observations also provide fundamental evidence and inspiration to propose a new RVE model for the prediction of UD-FRP properties.

In this paper, static tests were conducted to obtain the elastic properties of UD-FRP. Based on the random fiber inclination observed by micro-CT, a new micromechanical modeling method applicable to UD-FRP was proposed, where both the fiber position and inclination angle are randomly distributed. Based on the proposed modeling method, the distribution of fiber inclination angles included normal distribution and uniform distribution. The fiber diameter was assumed to be equal, as reported in the literature. Subsequently, the elastic properties observed in the experiments were compared with those predicted by the micromechanical model, which validated the proposed model. Finally, the proposed micromechanical model was used to investigate the effects of fiber volume fraction and maximum fiber inclination angle on elastic properties. The proposed micromechanical model with randomly inclined fibers helps to more accurately predict the elastic properties of UD-FRP, especially their transverse elastic properties.

## 2. Experiment

In this section, static tests were conducted to obtain the elastic properties of UD-FRP, and then micro-CT scanning technology was used to observe the fiber arrangement in UD-FRP.

### 2.1. Specimen Preparation

**Figure 1:**
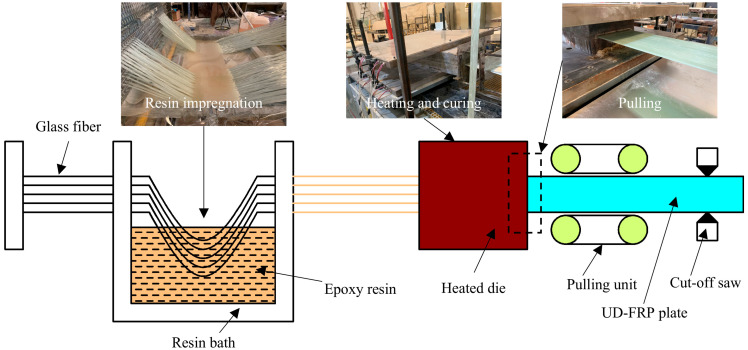
Schematic diagram of the pultrusion process.

**Figure 2:**
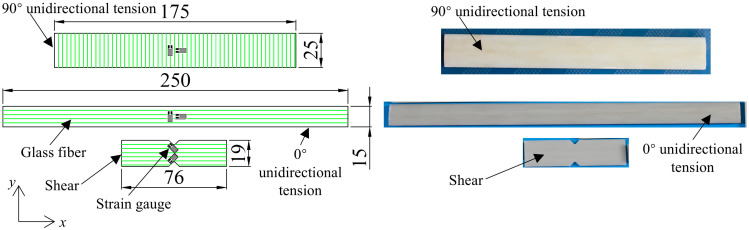
Specimen size and internal fiber structure.

Unidirectional glass fiber reinforced epoxy resin composites produced by the pultrusion process, as shown in Figure 1, were selected to carry out the static tests. The glass fibers were first guided into the resin bath for impregnation. Subsequently, the impregnated glass fibers were guided into the heated die and cured at a temperature of 90 °C for 30 min, then at 120 °C for 60 min, and finally at 150 °C for 40 min. Lastly, the cured UD-FRP plate was pulled out by the pulling unit. All specimens for static testing were cut from the same UD-FRP plate. The specimen dimensions and the fiber structure are shown in Figure 2, and its component properties are shown in Table 1. The epoxy in the form of MERICAN 3222H was purchased from SINO POLYMER Co., Ltd. in Shanghai, China, and its elastic modulus and Poisson’s ratio were 3.01 GPa and 0.38, respectively. The glass fiber in the form of EDR480-T910 was purchased from Taishan Glass Fiber Co., Ltd. in Taian, China, and its elastic modulus and Poisson’s ratio were 73.00 GPa and 0.22, respectively.

**Figure 3:**
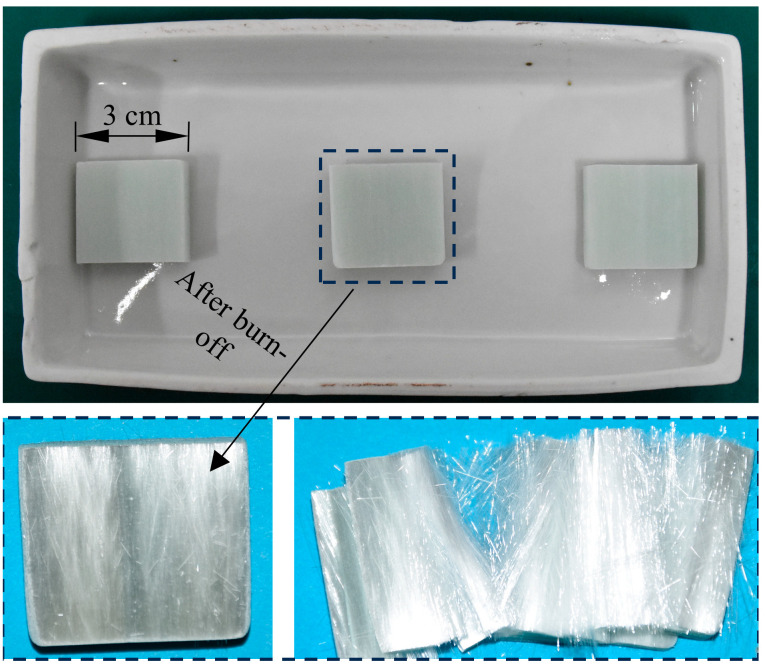
Burn-off tests of unidirectional fiber reinforced polymer (UD-FRP) composites.

The UD-FRP was manufactured by the pultrusion process, and the specimens tested were cut from the same pultruded sheet. The burn-off tests were carried out on the UD-FRP specimens to obtain their fiber volume fraction according to ASTM D 3171 [23], as shown in Figure 3. The burn-off test results are shown in Table 2, and the average fiber volume fraction is 62.96%.

### 2.2. Static Tests

**Figure 4:**
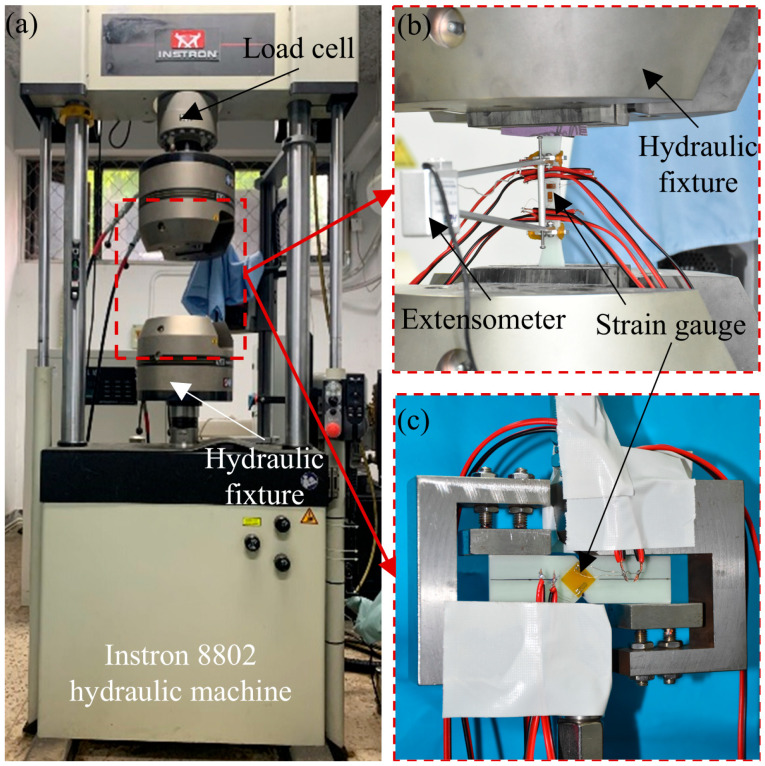
Static test setup: (**a**) Instron 8802 hydraulic machine; (**b**) tensile test; and (**c**) shear test.

**Figure 5:**
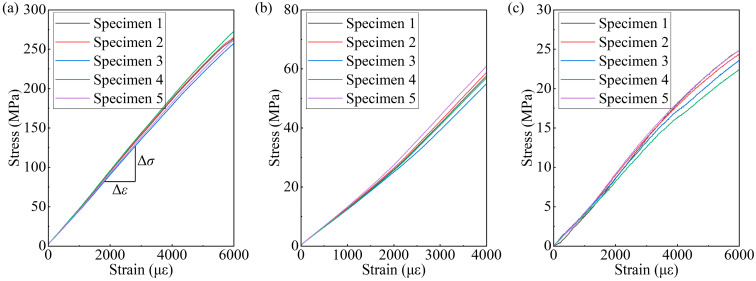
Stress-strain curves of static tests: (**a**) 0° unidirectional tension; (**b**) 90° unidirectional tension; and (**c**) shear.

Tensile and shear static tests were carried out on UD-FRP according to ASTM D 3039 [24] and ASTM D 5379 [25], and the number of specimens for each loading was 5 [26,27]. The static test setup is shown in Figure 4. All static tests were carried out using an Instron 8802 tensile testing machine, as shown in Figure 4a. The load applied to the specimen was measured by a load cell, as shown in Figure 4a. The deformation of the specimen was measured by strain gauges, as shown in Figure 4b,c, and the strain gauges on each face of the specimen were attached as shown in Figure 2. The 0/90° corner T rosette strain gauges were attached to both surfaces of the tensile specimen to measure the average axial strain and Poisson’s ratio, and −45/45° corner L rosette strain gauges were attached to both surfaces of the shear specimen to measure the shear strain. The strain gauge in the form of BF120-3AA (11) N6-F-X1-V2, supplied by Hualanhai Co., Ltd. in Dongguan, China, and had a resistance of 120 ohms. The displacement rate of the fixture was 2 mm‧min^−1^. The average stress-strain curves are shown in Figure 5, and the elastic modulus is defined by the slope of the initial linear portion of the stress-strain curve.

### 2.3. Microscopic Observation

**Figure 6:**
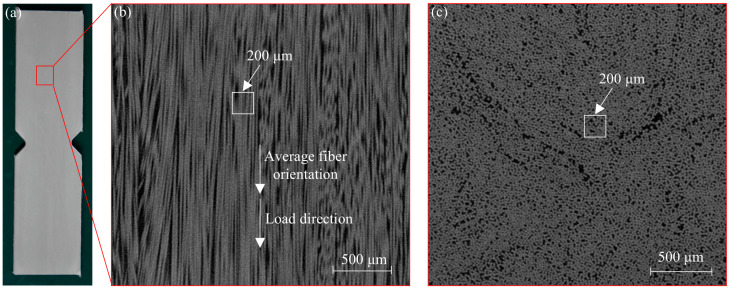
Microscopic computed tomography (CT) results: (**a**) specimen; (**b**) fiber orientation; and (**c**) fiber distribution in the cross section.

A cubic sample with a side length of 2.5 mm was cut from the specimen shown in Figure 6a for micro-CT. The gray-scale images obtained by micro-CT scanning parallel to and perpendicular to the fiber direction are shown in Figure 6b,c, respectively. As seen in Figure 6b, the fibers in the UD-FRP are not parallel to each other, and there is a slight random inclination of all the fibers. The fibers are randomly distributed in the cross-section, as shown in Figure 6c. It is noted that the RVE model represents only the overall and average distribution pattern of fibers in the UD-FRP [28,29,30].

## 3. Micromechanical Model Predictions

In this section, micromechanical models were used to calculate the elastic properties of UD-FRP. Specifically, appropriate algorithms were first used to generate randomly inclined fibers observed by micro-CT, and then these fibers were imported into the finite element software to calculate the elastic properties of UD-FRP.

### 3.1. Generation of Randomly Inclined Fibers

**Figure 7:**
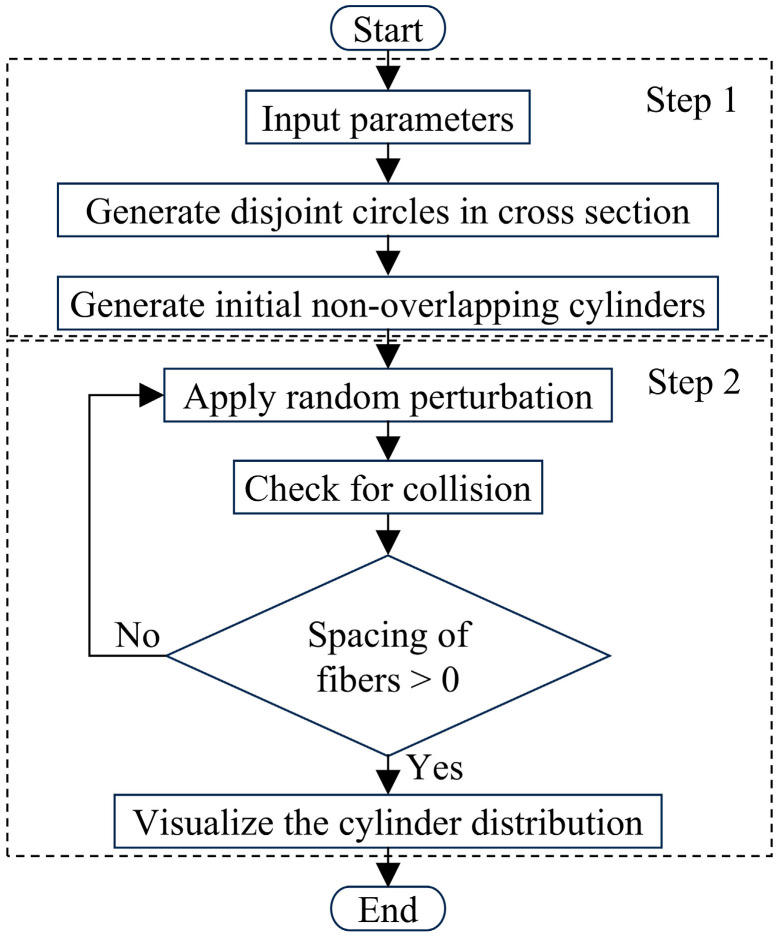
Generating randomly inclined fibers.

**Figure 8:**
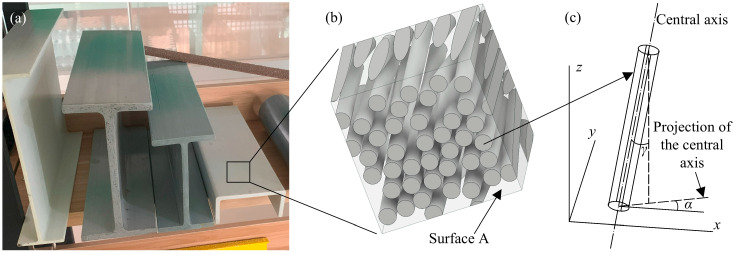
Inclined fibers in the representative volume element (RVE); (**a**) FRP profile; (**b**) the RVE; (**c**) single fiber.

As shown in Section 2.3, there is a random inclination of fibers in UD-FRP, so it is necessary to consider this random inclination in the RVE model and to investigate its effect on the prediction of elastic properties. The proposed method for generating randomly inclined fibers combines random perturbation, local search, and iterative optimization algorithms, as shown in Figure 7. This method includes two main steps: generating randomly distributed fibers in the fiber cross-section and applying random inclination perturbation to the fibers. Specifically, the side lengths, fiber volume fraction, and fiber diameters of the square RVE are first entered. Note that it has been shown that the diameter distribution of the fibers has little effect on the property prediction [11], so it is assumed in this paper that all fibers have the same diameter. Subsequently, randomly distributed circles are generated on the surface A as shown in Figure 8a,b, all of which do not intersect. An RVE with fibers parallel to each other can be generated based on these randomly distributed circles. Lastly, random inclination perturbation is applied to the fibers in this RVE, where the inclination angle *γ* is defined as the angle between the fiber axis and the *z*-axis, as shown in Figure 8c. The angle between the projection line of the fiber axis on the *xy*-plane and the *x*-axis is defined as *α*, which takes the range of −180°~180°. When *α* is less than 0, *γ* takes a negative value, and when *α* is greater than 0, *γ* takes a positive value. The fiber spacing is determined to be greater than 0 after all perturbations. Accordingly, the geometric model of RVE can be drawn using the above method, as shown in Figure 8b.

**Figure 9:**
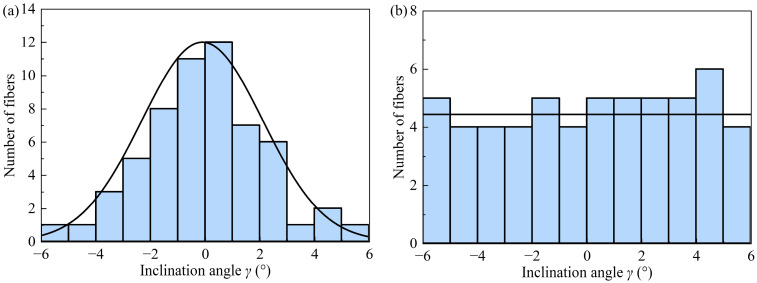
Distribution histogram of fiber inclination for fiber volume fraction of 63% and maximum inclination angle of 6°; (**a**) normal distribution; and (**b**) uniform distribution.

An RVE generated by the above method is shown in Figure 8b, where the side length is 200 μm, the fiber diameter is 24 μm, and the fiber volume fraction is 63%. According to reference [21], the maximum fiber inclination angle is 6° when the fiber volume fraction is around 63%. The fiber inclination that is normally distributed within this RVE is shown in Figure 9a. The mean and standard deviation of this normal distribution are −0.10° and 2.22°, respectively. In order to demonstrate that the method proposed in this paper can generate various fiber inclination distributions, uniformly distributed fiber inclinations are also generated, as shown in Figure 9b. The mean and standard deviation of this uniform distribution are 0.12° and 3.45°, respectively. In Figure 9, the maximum inclination angle for both the normal distribution and uniform distribution is 6°.

### 3.2. Finite Element Model of RVE

After obtaining the geometrical model of RVE by the above method, the equivalent properties of UD-FRP can be predicted by combining with the finite element software. In order to impose periodic boundary conditions (PBCs) on the RVE, Altair Hypermesh is used to generate periodic boundary meshes on the surface of the RVE. Specifically, all the curves on surface A in Figure 8b are projected onto its symmetric surface A’, and then the mesh on surface A’ is generated. Finally, the mesh on surface A’ is projected to face A. The periodic meshes on the remaining two sets of symmetry surfaces can be obtained in the same way. The pre-processed RVE model is imported into the ABAQUS 2021 to impose PBCs [11,31,32].

**Figure 10:**
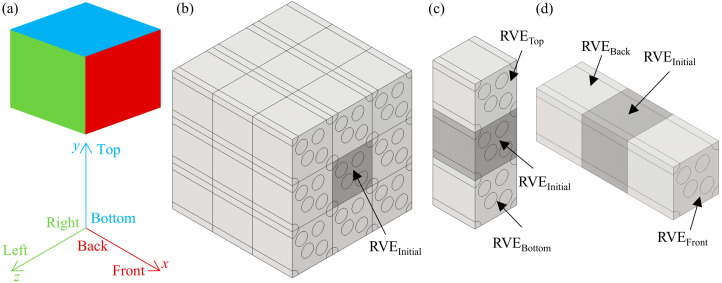
Schematic diagram of PBCs; (**a**) definition of the RVE symmetry planes; (**b**) FRP reconstructed from RVEs; (**c**) top and bottom boundary conditions for the calculation of *E*_11_; and (**d**) front and back boundary conditions for the calculation of *E*_11_.

The PBCs are imposed in ABAQUS 2021 by constraining the displacement of the RVE surface using equations. Note that the subscripts 1, 2, and 3 correspond to the *x*, *y*, and *z* axis directions, respectively. The PBCs for *E*_11_ and *G*_23_ are given by Equations (1) and (2), respectively [33,34], as follows:

(1a) $$X_{\mathrm{Front}}-X_{\mathrm{Back}}=X_{\mathrm{AssignedValue}}$$

(1b) $$X_{\mathrm{Top},\;\mathrm{Left}}-X_{\mathrm{Bottom},\;\mathrm{Right}}=0$$

(1c) $$Y_{\mathrm{Front},\;\mathrm{Top},\;\mathrm{Left}}-Y_{\mathrm{Back},\;\mathrm{Bottom},\;\mathrm{Right}}=0$$

(1d) $$Z_{\mathrm{Front},\;\mathrm{Top},\;\mathrm{Left}}-Z_{\mathrm{Back},\;\mathrm{Bottom},\;\mathrm{Right}}=0$$

(2a) $$X_{\mathrm{Front},\;\mathrm{Top},\;\mathrm{Left}}-X_{\mathrm{Back},\;\mathrm{Bottom},\;\mathrm{Right}}=0$$

(2b) $$Y_{\mathrm{Left}}-Y_{\mathrm{Right}}=Y_{\mathrm{AssignedValue}}$$

(2c) $$Y_{\mathrm{Top},\;\mathrm{Front}}-Y_{\mathrm{Bottom},\;\mathrm{Back}}=0$$

(2d) $$Z_{\mathrm{Front},\;\mathrm{Left}}-Z_{\mathrm{Back},\;\mathrm{Right}}=0$$

(2e) $$Z_{\mathrm{Top}}-Z_{\mathrm{Bottom}}=Z_{\mathrm{AssignedValue}}$$

where *X*, *Y*, and *Z* are the displacement components along the *x*, *y*, and *z* directions, respectively, and the subscript AssignedValue indicates the displacement applied in that direction. The surfaces perpendicular to the *x*-axis are defined as the front and back surfaces, the surfaces perpendicular to the *y*-axis are defined as the top and bottom surfaces, and the surfaces perpendicular to the *z*-axis are defined as the left and right surfaces, as shown in Figure 10a. The FRP on a macroscopic scale can be obtained by copying and translating the RVE in the different directions, as shown in Figure 10b. The PBCs used to calculate *E*_11_ are shown in Figure 10c,d. From Figure 10c, the RVE_top_, RVE_initial_, and RVE_bottom_ are equivalent, and the bottom surface of RVE_top_ overlaps with the top surface of RVE_initial_. Therefore, the top and bottom surfaces of each RVE maintain the same shape at all times, and when there is no load in the *y*-direction, the displacement of the top and bottom surfaces satisfies Equation (1b–d). As shown in Figure 10d, when a load is applied in the *x*-direction, the shapes of the front and back surfaces remain consistent, but there is a displacement difference between them due to the load. Their displacements satisfy Equation (1a,c,d), where *X*_AssignedValue_ is the displacement difference. The EasyPBC plugin for ABAQUS 2021 was used to apply PBCs to the RVE [35,36,37].

**Figure 11:**
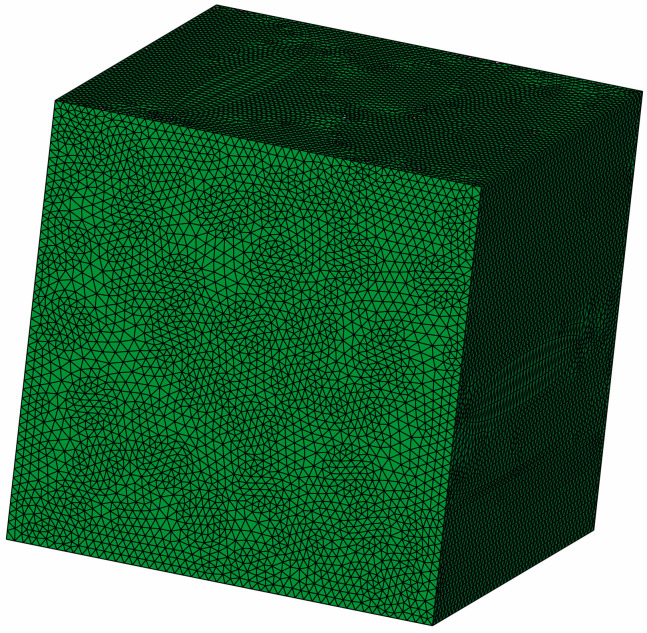
RVE model with finite element meshes.

It was assumed in the RVE model that the fiber-matrix interface is perfectly bonded, and the existence of the interphase zone was not considered. The assumption of perfect bonding is common when using RVE to study elastic behavior, as reported in the literature [33,38]. The material properties of the fibers and matrix were linear elastic. The ABAQUS/standard solver was utilized with a maximum incremental step of 100, and maximum and minimum incremental steps of 1 and 0.00005, respectively. The size of the used four-node tetrahedral element (C3D4) is about 3 μm, as shown in Figure 11 [39,40,41].

## 4. Results and Discussion

In this section, the elastic properties obtained from static tests were compared with those predicted by the micromechanical models. Subsequently, the validated model was used to investigate the effects of fiber volume fraction and maximum fiber inclination on the elastic properties.

### 4.1. Prediction of Elastic Properties

The mechanical properties of the matrix and fibers are the same as in Section 2.1. The fiber volume fraction in the RVEs with no fiber inclination, with fiber inclination normally distributed, and with fiber inclination uniformly distributed is 63%. According to the classical Hill’s homogeneous theory, the average stresses and strains of the RVE are given by [42,43,44]

(3) $$\bar{\sigma }=\frac{\sum_{i}V_{i}\sigma_{i}}{\sum_{i}V_{i}},\bar{\varepsilon }=\frac{\sum_{i}V_{i}\varepsilon_{i}}{\sum_{i}V_{i}}$$

where *σ_i_*, *ε_i_*, and *V_i_* are the stress, strain, and volume of the *i*th element, respectively. Subsequently, the equivalent elastic modulus of the RVE can be given by

(4) $$E_{\mathrm{RVE}}=\frac{\bar{\sigma }}{\bar{\varepsilon }}$$

Poisson’s ratio can be obtained from its definition combined with the average strain in the corresponding direction.

**Figure 12:**
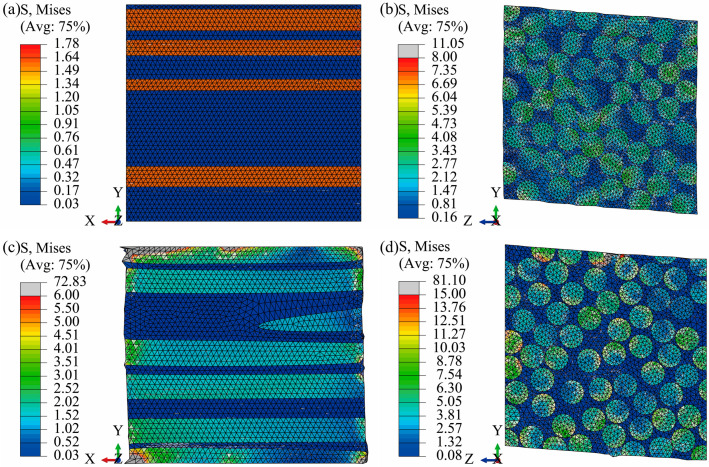
Stress cloud map for predicting RVE equivalent elastic modulus; (**a**) fibers are not inclined and the load is in the *x*-direction; (**b**) fibers are not inclined and the load is along the *yz*-plane; (**c**) fiber inclination is normally distributed and the load is in the *x*-direction; and (**d**) fiber inclination is normally distributed and the load is along the *yz*-plane.

The cloud map results used to predict the equivalent elastic properties of the RVE are shown in Figure 12. Note that the RVE cloud map results for a uniform distribution of fiber inclination are similar to those for a normal distribution. The loads in Figure 12a,c are along the *x*-axis direction as shown in Equation (1), and the RVE stress-strain results are used to predict *E*_11_, *ν*_12_, and *ν*_13_. It is noted that UD-FRP is a typically transversely isotropic material for which *ν*_12_ and *ν*_13_ should be equal. Meanwhile, the RVE stress-strain results under the in-plane shear load in the *yz* plane are used to predict *G*_23_. The elastic properties in the other directions can be calculated using stress and strain results similar to those in Figure 12. As shown in Figure 12, the stress in the fiber is significantly greater than that in the matrix, and the stress in the matrix is more uniform. When there is inclination in the fibers, a few regions of stress concentration appear at the fiber-matrix interface. Stress concentration is primarily determined by the fiber structure, meaning that smaller fiber spacing is more likely to cause stress concentration, as shown in Figure 12b,d. When fiber inclination is considered, the fiber spacing may be smaller. However, stress concentration in small regions does not affect the RVE calculation results, because predicting elastic properties requires the stress and strain of all elements, as shown in Equations (3) and (4). The experimental and predicted elastic properties are listed in Table 3. Moreover, the experimental 0° tensile, 90° tensile, and shear ultimate strengths were 1023.60 MPa, 65.47 MPa, and 76.25 MPa, respectively.

It is found that the difference between the predictions of *E*_11_, *ν*_12_, and *ν*_23_ predicted by the RVE model with and without fiber inclination is not significant. However, the *E*_22_ (*E*_33_) predicted by RVE without fiber inclination and with normally distributed fiber inclination are 11.45% and 0.30% lower than the experimental results, respectively. Moreover, the experimental value of *G*_31_ (*G*_21_) lies between the predicted results of RVE without fiber inclination and with normally distributed fiber inclination, and the predicted result of RVE without fiber inclination is 6.39% smaller than the experimental result. The *G*_23_ predicted by RVE with normal and uniform fiber inclination is 25.59% and 21.56% larger than that predicted by RVE without fiber inclination, respectively.

**Figure 13:**
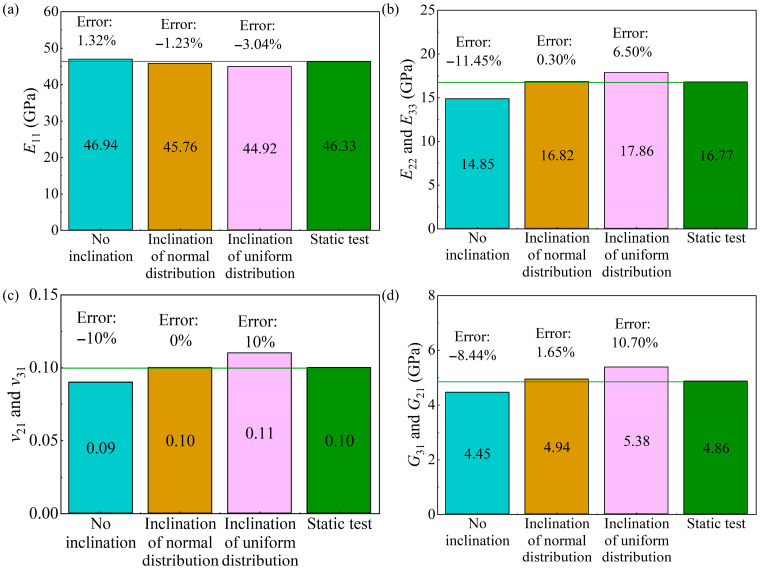
Relative errors of predicted results from micromechanical models; (**a**) *E*_11_; (**b**) *E*_22_ and *E*_33_; (**c**) *ν*_21_ and *ν*_31_; and (**d**) *G*_31_ and *G*_21_.

The relative error of the predicted elastic properties is shown in Figure 13. Note that all RVE models can accurately predict Poisson’s ratios *v*_12_ and *v*_13_, as shown in Table 3. As seen from Figure 13a, the Young’s modulus *E*_11_ predicted by all RVE models showed good consistency with the test results, with the relative error of about 2%. The *E*_22_ and *E*_33_ predicted by the RVE with normally distributed fiber inclination were closest to the experimental result, with a relative error of only 0.30%, while the relative error of the RVE without fiber inclination was 11.45%, as shown in Figure 13b. When considering the normal distribution of fiber inclination, the RVE can accurately predict Poisson’s ratios *v*_21_ and *v*_31_, as shown in Figure 13c. Similarly, when the fiber inclination was normally distributed, the shear moduli *G*_31_ and *G*_21_ predicted by RVE were closest to the test results, with a relative error of only 1.65%, as shown in Figure 13d.

Fiber structure significantly affects the prediction of equivalent properties by RVE, especially for transverse properties. Accordingly, it is necessary to consider fiber inclination in the RVE model in order to predict the mechanical properties of UD-FRP more accurately. Although the proposed RVE model can accurately predict the elastic properties of UD-FRP, the model still has potential limitations. The proposed RVE modeling method currently cannot account for initial defects such as voids and fiber waviness in UD-FRP. Moreover, the current RVE model does not consider the fiber-matrix interface. The aforementioned potential limitations have little impact on the prediction of UD-FRP elastic properties, but may significantly affect the prediction of strength, viscoelastic behavior, and fatigue behavior.

### 4.2. Effect of Fiber Volume Fraction on Elastic Properties

**Figure 14:**
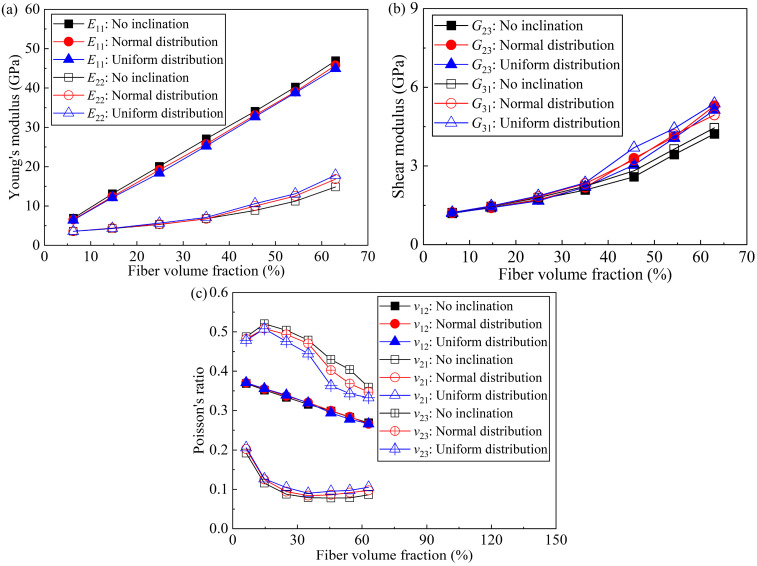
Effect of fiber volume fraction on RVE elastic properties for fibers without inclination, fibers with inclination normally distributed, and fibers with uniform distribution, respectively; (**a**) Young’s modulus; (**b**) shear modulus; and (**c**) Poisson’s ratio.

The fiber volume fraction has a significant impact on the elastic [45], tribological [46], and fatigue [47] properties of FRP. Therefore, it is valuable to use the new micromechanical model proposed in this paper to study the effect of fiber volume fraction on elastic properties. The predicted elastic properties using RVEs with fiber volume fractions of 5%, 15%, 25%, 35%, 45%, 55% and 65% are presented to investigate the effect of fiber volume fractions on the elastic properties of UD-FRP. The elastic properties of RVEs with no fiber inclination, with fiber inclination normally distributed, and with fiber inclination uniformly distributed are also compared, as shown in Figure 14. The maximum inclination angle is 6°. Due to the perturbation algorithm for generating inclined fibers, the actual fiber volume fractions are slightly different from the target values and are 5.65%, 14.95%, 24.88%, 34.95%, 45.57%, 55.63% and 63.00%. The predicted *E*_11_ increases linearly with fiber volume fractions, and the prediction considering fiber inclination is slightly smaller than that without fiber inclination, as shown in Figure 14a. Moreover, the difference in predicted elastic properties between RVE models with and without fiber inclination increases with increasing fiber volume fraction. Specifically, the RVE-predicted *E*_11_ without fiber inclination increased from 6.89 GPa to 40.27 GPa as the fiber volume fraction increased from 5% to 55%. Moreover, the difference between the RVE-predicted *E*_11_ with the normally-distributed fiber inclination and the one without inclination was only 3.06% when the fiber volume fraction was 55%.

The predicted *E*_22_ (*E*_33_) has similar trends, except that it increases nonlinearly with fiber volume fractions. Uniformly distributed fiber inclinations lead to more significant deviations in predicted Young’s modulus compared to normally distributed inclinations. The difference between *E*_22_ (*E*_33_) predicted by the RVE without fiber inclination and with normally distributed fiber inclination was larger than that of *E*_11_; For *E*_22_ (*E*_33_), this difference was 11.99% when the fiber volume fraction was 55%.

The predicted trends of the shear moduli *G*_23_ and *G*_31_ (*G*_21_) with increasing fiber volume fraction are similar to that of the Young’s modulus *E*_22_ (*E*_33_), as shown in Figure 14b, and this nonlinear increase in shear modulus with fiber volume fraction was reported in the literature [48,49]. When the fiber volume fraction was 55%, the *G*_23_ predicted by RVE with normally distributed fiber inclination was 19.49% larger than that without considering fiber inclination.

From Figure 14c, *ν*_12_ (*ν*_13_) decreases linearly with fiber volume fractions and is not affected by fiber inclination. The predicted *ν*_21_ (*ν*_31_) first decreases and then remains almost constant (about 0.10) with increasing fiber volume fractions and is larger when fiber inclination is considered. Similarly, the effect of the uniformly distributed fiber inclination is more significant than that of the normal distribution. The predicted *ν*_23_ increases slightly and then decreases rapidly with fiber volume fractions, and is lower when the fiber inclination is considered.

It can be seen that fiber volume fractions have a significant effect on the elastic properties. The fiber inclination angle in the RVE model has different effects on the prediction of elastic properties and has a significantly greater effect on the transverse elastic properties than on the longitudinal elastic properties. The reason for this is that the properties of UD-FRP are mainly determined by the internal unidirectional fibers, and the projection of the inclined fibers in the transverse direction is much larger than that of the non-inclined fibers. However, the projection in the longitudinal direction is little changed compared to that of the non-inclined fibers.

### 4.3. Effect of Maximum Fiber Inclination on Elastic Properties

**Figure 15:**
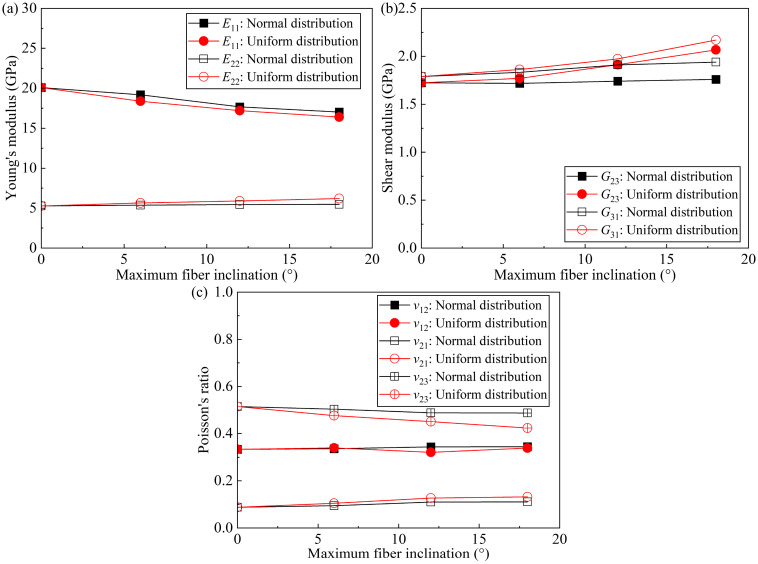
Effect of maximum fiber inclination on RVE elastic properties for fibers without inclination, fibers with inclination normally distributed, and fibers with uniform distribution, respectively; (**a**) Young’s modulus; (**b**) shear modulus; and (**c**) Poisson’s ratio.

Fiber inclination has a significant effect on the prediction of elastic properties, and the effect at a maximum fiber inclination of 6° is shown in Section 4.1 and Section 4.2. It is noted that the fiber inclination angle decreases with fiber volume fractions due to geometric constraints. Therefore, in order to further investigate the effect of maximum fiber inclination on elastic properties, RVEs with lower fiber volume fractions are selected. Specifically, the RVEs with 25% fiber volume fractions are selected with maximum inclination angles of 0°, 6°, 12°, and 18°, and the distributions of fiber inclination angles include normal and uniform distributions. The predicted elastic properties are shown in Figure 15.

The predicted *E*_11_ decreases linearly with the increasing maximum fiber inclination angle, with a more pronounced reduction observed for uniformly distributed inclination, as shown in Figure 15a. Moreover, when the maximum fiber inclination is 18°, the *E*_11_ of RVE with normally and uniformly distributed fiber inclination is 15.31% and 18.45% lower than that without inclination, respectively. The predicted *E*_22_ (*E*_33_) increases linearly with the maximum fiber inclination angle. When the maximum fiber inclination is 18°, the *E*_22_ (*E*_33_) of RVE with normally and uniformly distributed fiber inclination is 3.55% and 17.23% higher than that without inclination, respectively.

The predicted *G*_23_ and *G*_31_ (*G*_21_) increase with increasing maximum fiber inclination, as shown in Figure 15b. When the maximum fiber inclination is 18°, the *G*_23_ of RVE with normally and uniformly distributed fiber inclination is 2.00% and 19.89% higher than that without inclination, respectively. In the case of *G*_31_ (*G*_21_), they are 8.40% and 21.23% higher, respectively.

The presence of fiber inclination has little effect on the prediction of *ν*_12_ (*ν*_13_), as shown in Figure 15c. The predicted *ν*_21_ (*ν*_31_) and *ν*_23_ increase and decrease linearly with increasing maximum fiber inclination, respectively. Moreover, when the maximum fiber inclination is 18°, the *ν*_21_ (*ν*_31_) of RVE with normally and uniformly distributed fiber inclination is 26.40% and 50.57% higher than that without inclination, respectively; In the case of *ν*_23_, they are 5.33% and 17.72% lower, respectively.

It can be seen that the maximum fiber inclination has a significant effect on the prediction of all the elastic properties of the RVE (except for *ν*_12_ (*ν*_13_)), and these effects increase with the maximum fiber inclination. The maximum fiber inclination increases the projection of the fibers in the cross-section and so affects the elastic properties of the RVE.

### 4.4. Interactive Effects of Fiber Volume Fractions and Maximum Fiber Inclination on Elastic Properties

After demonstrating the effects of the individual parameters of fiber volume fractions or maximum fiber inclination, it is necessary to further investigate their interactive effects.

**Figure 16:**
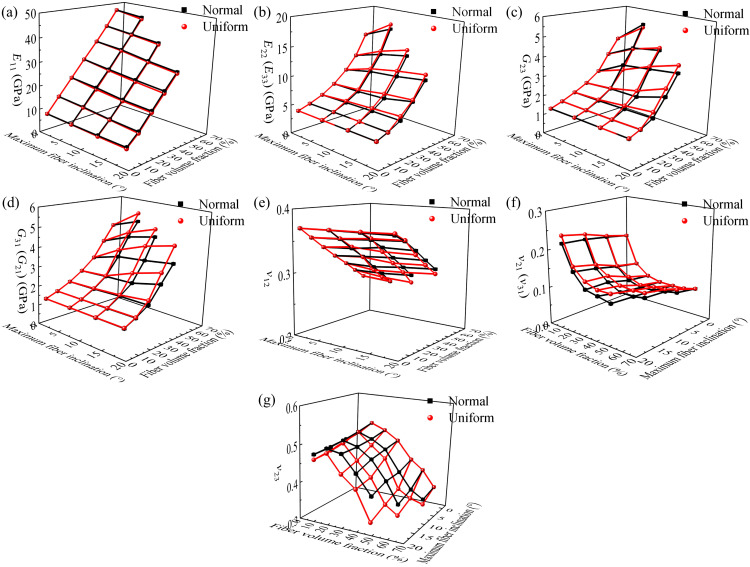
Effect of fiber volume fraction and maximum fiber inclination on RVE elastic properties for fibers without inclination, fibers with inclination normally distributed and fibers with uniform distribution, respectively; (**a**) *E*_11_; (**b**) *E*_22_ (*E*_33_); (**c**) *G*_23_; (**d**) *G*_31_ (*G*_21_); (**e**) *ν*_12_ (*ν*_13_); (**f**) *ν*_21_ (*ν*_31_); and (**g**) *ν*_23_.

The effect of fiber volume fractions and maximum fiber inclination on the elastic properties of the RVE is shown in Figure 16. The maximum fiber inclinations in all RVE models include 6°, 12°, and 18°, and there are 5%, 15%, 25%, 35%, 45%, 55% and 65% fiber volume fractions at each fiber maximum inclination. Note that the algorithm, as shown in Figure 7, does not converge when the fiber volume fraction is 65% and the maximum fiber inclination is 12° or 18°. This may be due to the geometric constraints in the RVE, where the fiber maximum inclination decreases with fiber volume fraction.

From Figure 16, the effects of fiber volume fraction and maximum inclination angle on the elastic properties are independent, and the trends of the elastic properties are similar to those in Figure 14 and Figure 15. The difference between the elastic properties of RVEs with normally and uniformly distributed fiber inclination increases with fiber volume fraction or maximum inclination angle. For *G*_31_ (*G*_21_), this difference reaches 24.64% when the fiber volume fraction was 45% and the maximum fiber inclination angle was 18°. For *ν*_23_, this difference reaches 21.79% when the fiber volume fraction was 45% and the maximum fiber inclination angle was 18°.

Moreover, the fiber volume fraction and the maximum inclination angle affect the longitudinal properties (*E*_11_ and *ν*_12_ (*ν*_13_)) linearly, while the effect on the transverse properties becomes significantly nonlinear.

## 5. Conclusions

In this paper, the random inclination of fibers is considered in the RVE model, where the distribution of fiber inclination includes normal and uniform distributions. After verifying the proposed RVE model with experiments, the effects of fiber volume fraction and maximum fiber inclination angle on the elastic properties are investigated. Several important conclusions are summarized as follows:

(1) The developed RVE model can accurately predict the elastic properties of UD-FRP, and the experimental results lie between those predicted by RVE with no fiber inclination and with fiber inclination normally distributed.
(2) The elastic properties *E*_11_ and *ν*_12_ increase and decrease linearly with fiber volume fraction, respectively. The *E*_22_ (*E*_33_), *G*_23,_ and *G*_31_ (*G*_21_) increase nonlinearly with fiber volume fraction. The *ν*_21_ (*ν*_31_) decreases and then stays constant with fiber volume fraction. The *ν*_23_ increases slightly and then decreases rapidly and linearly with fiber volume fraction.
(3) The elastic properties *E*_11_ and *ν*_23_ decrease linearly with maximum fiber inclination, while *E*_22_ (*E*_33_) and *ν*_21_ (*ν*_31_) increase linearly. The *G*_23_ and *G*_31_ (*G*_21_) increase nonlinearly with maximum fiber inclination. The maximum fiber inclination has no effect on *ν*_12_.
(4) Fiber inclination in the RVE has a significantly greater effect on transverse elastic properties than on longitudinal elastic properties. Moreover, for the prediction of elastic properties, uniformly distributed inclination has a more pronounced effect than normally distributed inclination.

## Figures and Tables

**Table 1:** Material properties of epoxy and fibers.

Epoxy Precursor			Anhydride Curing Agent			Mixture (1:1 Mass Ratio)		Glass Fiber		
Viscosity (cps)	Density (g·cm^−3^)	Epoxy Value (mol·(100·g)^−1^)	Viscosity (cps)	Density (g·cm^−3^)	Acid Value (mgKOH·g^−1^)	Viscosity (cps)	Density (g·cm^−3^)	Diameter (μm)	Density (g·cm^−3^)	Breaking force (N)
9500	1.15	0.55	150	1.15	590	550	1.15	25	2.54	24

**Table 2:** Burn-off test results of UD-FRP.

Specimens	Volume (cm^3^)		Fiber Volume Fraction (%)
	Before Burn-Off	After Burn-Off	
1	1.563	0.984	62.96
2	1.552	0.978	63.02
3	1.557	0.979	62.90

**Table 3:** The elastic properties from the static tests and RVE model with 65% fiber volume fraction for different fiber inclination distributions.

Maximum Inclination Angle (°)	Inclination Distribution	*E*_11_ (GPa)	*ν*_12_ (*ν*_13_)	*E*_22_ (*E*_33_) (GPa)	*ν*_21_ (*ν*_31_)	*ν*_23_ (*ν*_32_)	*G*_23_ (GPa)	*G*_31_ (*G*_21_) (GPa)
0	/	46.94	0.27	14.85	0.09	0.36	4.22	4.45
6	Normal distribution	45.76	0.27	16.82	0.10	0.35	5.30	4.94
6	Uniform distribution	44.92	0.27	17.86	0.11	0.33	5.13	5.38
Static test		46.33	0.27	16.77	0.10	/	/	4.86

## Data Availability

The original contributions presented in this study are included in the article. Further inquiries can be directed to the corresponding author.

## Abbreviations

The following abbreviations are used in this manuscript:

RVE	Representative volume element
CT	Computed tomography
FRP	Fiber-reinforced polymer
UD-FRP	Unidirectional FRP
FE	Finite element
PBC	Periodic boundary condition
